# Developing an Undergraduate Ultrasound Curriculum: A Needs Assessment

**DOI:** 10.7759/cureus.1720

**Published:** 2017-09-28

**Authors:** Jordan Stone-McLean, Brian Metcalfe, Gillian Sheppard, Justin Murphy, Holly Black, Heather McCarthy, Adam Dubrowski

**Affiliations:** 1 Faculty of Medicine, Memorial University of Newfoundland; 2 Orthopaedic Surgery, Memorial University of Newfoundland; 3 Emergency Medicine, Memorial University of Newfoundland; 4 Emergency Medicine, Pediatrics, Memorial University of Newfoundland

**Keywords:** ultrasound, pocus, undergraduate medical education, teaching, needs assessment, point-of-care ultrasound, curriculum development

## Abstract

Background

The introduction of ultrasound into the undergraduate medical school curriculum is gaining momentum in North America. At present, many institutions are teaching ultrasound to undergraduate medical students using a traditional framework designed to instruct practicing clinicians, or have modeled the curriculum on other universities. This approach is not based on educational needs or supported by evidence.

Methods

Using a descriptive, cross-sectional survey of stakeholder groups, we assessed the perceived relevance of various ultrasound skills and the attitude towards implementing an undergraduate ultrasound curriculum at our university.

Results

One hundred and fifty survey respondents representing all major stakeholder groups participated. All medical students, 97% of residents and 82% of educators agreed that the introduction of an ultrasound curriculum would enhance medical students' understanding of anatomy and physiology. All clinical medical students and residents, 92% of preclinical medical students, and 82% of educators agreed that the curriculum should also include clinical applications of ultrasound. Participants also indicated their preferences for specific curriculum content based on their perceived needs.

Conclusion

An integrated undergraduate ultrasound curriculum composed of specific preclinical and clinical applications was deemed appropriate for our university following a comprehensive needs assessment. Other universities planning such curricula should consider employing a needs assessment to provide direction for curriculum need and content.

## Introduction

Ultrasound has become a desired and increasingly common addition to medical school curricula across North America [1-5]. To date, few comprehensive undergraduate ultrasound programs have been described in the literature, and even fewer are based on educational needs, grounded in educational theory, or supported by evidence [1-4]. These skills have been taught to medical learners using existing or adapted courses originally designed for practicing clinicians, or have been modeled after more established programs at other institutions.

There is currently no accepted standard undergraduate ultrasound curriculum in North America, and there are no described needs assessments of all stakeholders to inform the content and structure of such curriculum. Without this knowledge, medical students may continue to learn curriculum that is irrelevant, inappropriate for their level of training, or impractical for future medical practice while consuming university resources.

Our goal is to develop a comprehensive, evidence-based undergraduate ultrasound curriculum based on the perceived needs of all stakeholders. Our curriculum development will incorporate accepted learning theory and follow the Medical Research Council of Canada’s Framework for Developing and Evaluating Complex Interventions, the first stage of which is addressed in this study [6].

## Materials and methods

A descriptive, cross-sectional survey of stakeholder groups was conducted to assess the perceived relevance of various ultrasound skills, and the attitude towards implementing an undergraduate ultrasound curriculum at our university. There were 846 participants invited, including all 276 medical students, 270 medical residents, and 300 clinical and basic medical science educators from our university. Preclinical medical students were given a short briefing prior to a scheduled lecture and encouraged to participate. Clinical medical students, residents and faculty were not available to be contacted in a similar way. All participants received a reminder email after three weeks, and the survey was closed after a six-week period.

The survey was hosted by FluidSurveys^TM^ and questions were informed by the available body of research on ultrasound in medical education and relevant ultrasound topics. Participants were asked to answer questions using a five-point Likert scale ranging from “strongly agree” to “strongly disagree”. Basic demographic information was also collected.

Data was analyzed using Microsoft Excel (Microsoft Office 2011). Each group (preclinical medical students, clinical medical students, medical residents and educators) was analyzed for frequency of the five possible responses. The responses “agree” and “strongly agree” were grouped to give the percentage of respondents that were generally in favour of the statement in question. The responses “disagree” and “strongly disagree” were treated in the same manner. The dataset was de-identified prior to analysis.

In an attempt to interpret the increased frequency of “neutral” responses among medical school faculty, a focus group was conducted. A total of 12 faculty members agreed to participate in a focus group. Participants were asked to expand on their responses, particularly the questions that received the lowest rate of positive responses from faculty members. Faculty were also given an opportunity to voice any ideas, concerns, or potential barriers to implementation of an undergraduate ultrasound curriculum. The focus group was audio recorded and transcribed for analysis.

An application to the local Health Research Ethics Authority was submitted, and our study was deemed exempt from full review.

## Results

One hundred and fifty-one participants completed the survey and their demographics are listed in Table 1. The combined response rate of all stakeholders invited to participate was 17.8%, and their survey responses are summarized in Table 2. Of all respondents, 76.7% had no formal training in ultrasound, and 70% reported not having access to ultrasound machines. Despite this, 78% of clinical medical students, 97% of residents, and 84% of staff physicians agreed that point-of-care ultrasound was relevant to their clinical practice.

**Table 2:** Participant Demographics by Sex, Age and Group n: number SD: Standard Deviation

Group	Males n (%)	Females n (%)	Mean age (SD)
Pre-clinical students	21 (14)	43 (28)	25.4 (2.8)
Clinical students	11 (7)	7 (5)	28.1 (4.2)
Residents	15 (10)	15 (10)	30.0 (3.8)
Educators	23 (15)	16 (11)	48.9 (9.4)
Total	70 (46)	81 (54)	32.8 (11.2)

**Table 1:** Needs Assessment Survey Summary n/a: not applicable

Question	Preclinical Students			Clinical Students			Residents			Educators		
	Agree (%)	Neutral (%)	Disagree (%)	Agree (%)	Neutral (%)	Disagree (%)	Agree (%)	Neutral (%)	Disagree (%)	Agree (%)	Neutral (%)	Disagree (%)
1. Bedside ultrasound is clinically relevant to my medical practice	n/a	n/a	n/a	77.8	22.2	0.0	96.6	3.3	0.0	84.2	10.5	5.3
2. Bedside ultrasound would augment medical students' understanding of anatomy and physiology	100.0	0.0	0.0	100.0	0.0	0.0	96.6	3.3	0.0	81.6	10.5	7.9
3. Learning head and neck anatomy could be improved by using bedside ultrasound	100.0	0.0	0.0	83.3	16.7	0.0	86.7	10.0	3.3	50.0	39.5	10.5
4. Learning abdominal anatomy could be improved by using bedside ultrasound	100.0	0.0	0.0	100.0	0.0	0.0	96.6	3.3	0.0	76.3	21.1	2.6
5. Learning pelvic anatomy could be improved by using bedside ultrasound	100.0	0.0	0.0	100.0	0.0	0.0	93.3	6.7	0.0	65.8	31.6	2.6
6. Medical students would benefit from learning clinical applications of bedside ultrasound	92.2	7.8	0.0	100.0	0.0	0.0	100.0	0.0	0.0	81.6	15.8	2.6
7. Residents would benefit from learning clinical applications of bedside ultrasound	92.2	7.8	0.0	100.0	0.0	0.0	100.0	0.0	0.0	94.7	5.3	0.0
8. Medical students would benefit from being competent in the following bedside ultrasound skills: Assessing volume status (IVC measurement)	89.1	6.3	4.7	83.3	16.7	0.0	60.0	30.0	10.0	50.0	28.9	21.1
9. Medical students would benefit from being competent in the following bedside ultrasound skills: Obtaining vascular access	90.6	4.7	4.7	94.4	5.6	0.0	90.0	3.3	6.7	73.7	13.2	13.2
10. Medical students would benefit from being competent in the following bedside ultrasound skills: Performing peripheral nerve blocks	87.5	7.8	4.7	66.7	16.7	16.7	66.7	23.3	10.0	23.7	50.0	26.3
11. Medical students should be competent in the diagnosis of the following conditions utilizing bedside ultrasound: Deep-vein thrombosis	95.3	1.6	3.1	66.7	33.3	0.0	56.7	33.3	10.0	34.2	36.8	28.9
12. Medical students should be competent in the diagnosis of the following conditions utilizing bedside ultrasound: Abdominal aortic aneurysm	95.3	1.6	3.1	94.4	5.6	0.0	66.7	23.3	10.0	50.0	31.6	18.4
13. Medical students should be competent in the diagnosis of the following conditions utilizing bedside ultrasound: Ectopic pregnancy	96.9	0.0	3.1	77.8	16.6	5.6	63.3	26.7	10.0	47.4	34.2	18.4
14. Medical students should be competent in the diagnosis of the following conditions utilizing bedside ultrasound: Cardiogenic shock	89.1	7.8	3.1	66.7	33.3	0.0	60.0	36.7	3.3	31.6	50.0	18.4
15. Medical students should be competent in the diagnosis of the following conditions utilizing bedside ultrasound: Thyroid masses	89.1	7.8	3.1	77.8	16.7	5.6	50.0	46.7	3.3	31.6	42.1	26.3
16. Medical students should be competent in the diagnosis of the following conditions utilizing bedside ultrasound: Intra-abdominal hemorrhage	92.2	4.7	3.1	77.8	16.7	5.6	80.0	16.7	3.3	50.0	39.5	10.5
17. Medical students should be competent in the diagnosis of the following conditions utilizing bedside ultrasound: Pneumothorax	93.8	3.1	3.1	66.7	33.3	0.0	73.3	26.7	0.0	47.4	39.5	13.2
18. Medical students should be competent in the diagnosis of the following conditions utilizing bedside ultrasound: Pleural effusion	93.8	3.1	3.1	83.3	16.7	0.0	76.7	23.3	0.0	55.3	31.6	13.2
19. My current practice would be enhanced if I had learned bedside ultrasound during my undergraduate medical training	n/a	n/a	n/a	100.0	0.0	0.0	90.0	10.0	0.0	57.9	28.9	13.2
20. Ultrasound guidance during invasive procedures, such as central venous catheter placement and paracentesis, would improve patient safety	95.3	4.7	0.0	100.0	0.0	0.0	100.0	0.0	0.0	89.5	7.9	2.6
21. The Faculty of Medicine at Memorial University would attract more applicants if its undergraduate curriculum included bedside ultrasound training	50.0	43.8	6.3	38.9	44.4	16.7	50.0	43.3	6.7	28.9	50.0	21.1
22. Memorial University should incorporate bedside ultrasound into its undergraduate medical school curriculum	98.4	1.6	0.0	94.4	5.6	0.0	90.0	10.0	0.0	71.1	23.7	5.3

All groups indicated that our university should incorporate ultrasound into its undergraduate medical school curriculum. Among educators, 71% identified a need for such curriculum, and 24% provided a neutral response. No medical learners opposed the potential new curriculum.

All groups agreed that using ultrasound as a teaching adjunct would help improve anatomy instruction. The medical trainee groups agreed more strongly than the educators. All medical students and 97% of residents believed that the use of ultrasound would improve teaching of abdominal anatomy compared to 76% of educators. Consensus was less strong with head and neck anatomy as 100% of preclinical students, 83% of clinical students, 87% of residents, and just 50% of educators thought that ultrasound would improve head and neck anatomy teaching, with 40% of educators being neutral. Similarly, almost all learners thought that ultrasound could augment learning of pelvic anatomy while only 56% of educators felt the same, with 31% being neutral.

There was support for learning the clinical applications of ultrasound at both the undergraduate and postgraduate level. Almost all medical learners indicated that clinical applications of ultrasound should be taught to medical students; 82% of educators agreed, and 16% provided neutral responses. Educator support for residents learning the clinical applications of bedside ultrasound was very high.

Support from medical learners was high for specific clinical applications of ultrasound at the undergraduate level but was mixed among educators with large numbers of neutral ratings. The only clinical application to receive strong support among educators was ultrasound-guided vascular access (74%). At least 50% of educators agreed that evaluation for pleural effusion, abdominal aortic aneurysm, intra-abdominal hemorrhage, and volume status were important to learn at the undergraduate level. For all other clinical applications, (deep-vein thrombosis, pneumothorax, cardiogenic shock, peripheral nerve blocks, ectopic pregnancy, and thyroid masses) educator disagreement was moderate, and a large number of educators were neutral.

Ninety-five percent of preclinical students, 100% of clinical students and residents, and 90% of educators indicated that ultrasound guidance during invasive procedures enhances patient safety.

The members of the focus group were asked why there were a higher number of neutral responses provided from the educator group for nearly all questions. Participants attributed the difference to the relatively large proportion of educators at our institution who are not trained in ultrasound and were unable to fully appreciate the potential applications.

Participants were also questioned on the attitudes of educators teaching medical students specific clinical applications of ultrasound. The focus group felt that peripheral nerve blocks are a higher-order procedure involving multiple skills. In addition, performing peripheral nerve blocks is limited to specific specialists, and may not be applicable to training generalists. The group did indicate that ultrasound would be valuable in teaching peripheral nerve anatomy. 

## Discussion

Medical schools have begun to incorporate ultrasound into their undergraduate programs. The appropriateness of such curriculum and its ability to meet the individual needs of each institution is usually not evidence based. Furthermore, extensive ultrasound curriculum integration is the exception in Canadian medical schools. Although 67% of medical schools surveyed in one Canadian study offered ultrasound instruction, half of these schools provided only one to five hours of ultrasound teaching, and 67% taught ultrasound in the final two years of medical school as part of a clinical rotation [7]. Similar findings are seen in the United States where 61% offered such instruction, with only 18% of those schools making it a priority [8].

Traditionally, point-of-care ultrasound curriculums have been designed for practicing clinicians to augment clinical decision making. Undergraduate curriculum designers should pivot this goal to focus on basic ultrasound skills, informing medical education, and enhancing physical examination to better align with this stage of medical education. There is a large body of evidence confirming that ultrasound enhances a medical student's understanding of anatomy and physiology and improves physical examination skills [9-18].

**Figure 1:**
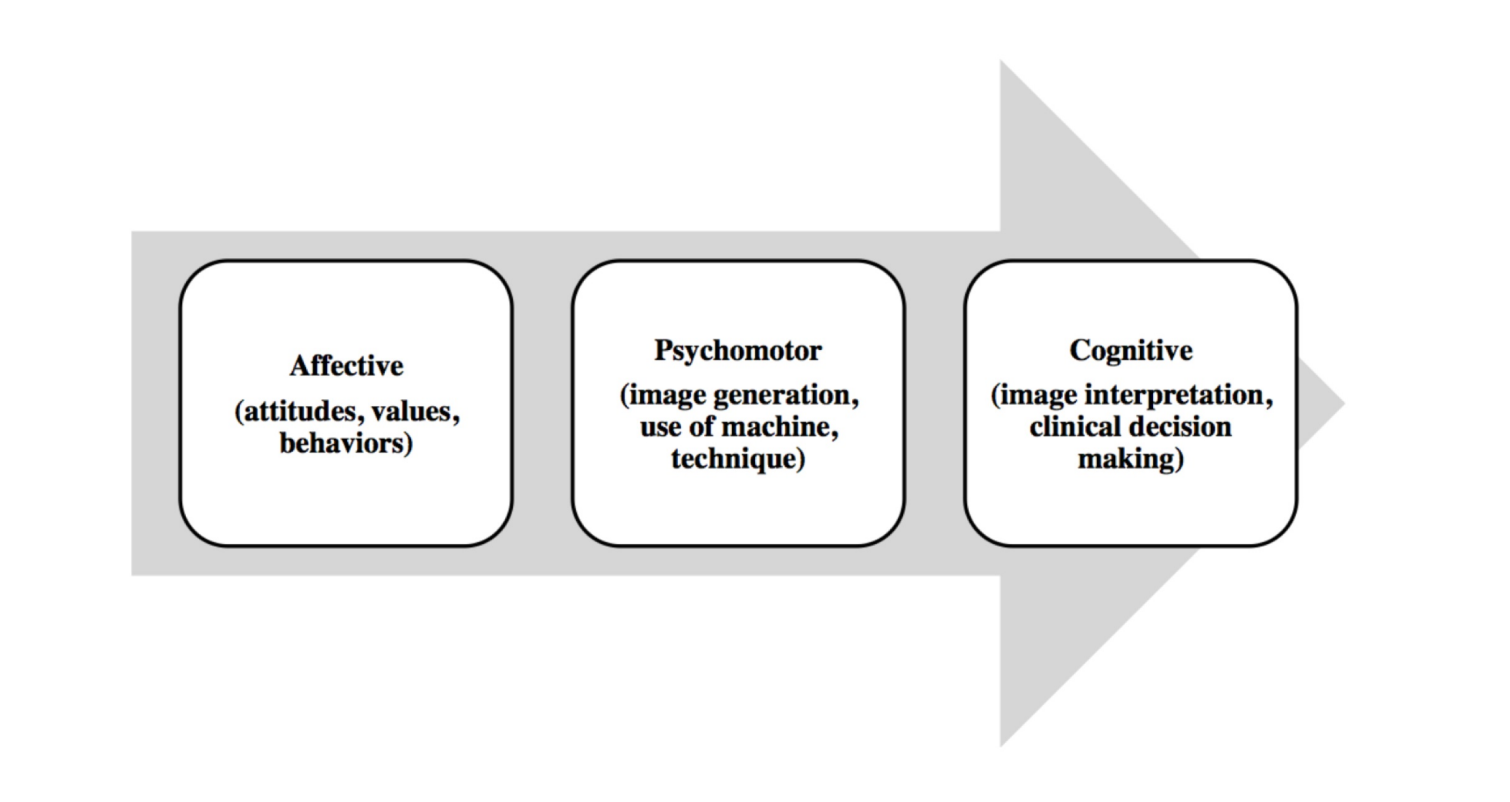
Educational theory underpinning undergraduate ultrasound curriculum (adapted from Bloom’s taxonomy of educational objectives) Adapted from Bloom’s taxonomy of educational objectives [20].

In learning theory, variability of practice predicts that practicing multiple variations of a skill has significant advantages over constant repetition of a skill, which allows for the development of a relationship between purposeful movement and specific outcomes [19]. Introduction of basic ultrasound concepts at the earliest stages of medical education allows for a diverse foundation of ultrasound-specific cognitive and motor skills that are later transferable to more demanding clinical applications preformed at a higher cognitive load. Figure 1, adapted from Bloom’s taxonomy of educational objectives, depicts a theoretical framework on which to build such curricula [20].

Learners and educators at our university consider undergraduate ultrasound curriculum to be important. There is a desire for an undergraduate ultrasound curriculum that complements anatomical, physiological, and clinical teaching. This involves a more extensive and integrated curriculum than what is currently being offered at most North American medical schools, as well as an earlier introduction into the curriculum. The highly positive response from medical learners is consistent with data available from the UK and the United States [9, 21].

Educators provided more neutral responses when surveyed about specific anatomical structures and physiology concepts they wished to see taught using ultrasound. This was in stark contrast to their enthusiasm expressed when surveyed about anatomy and physiology in general. Our focus group confirmed that inexperience using ultrasound as a teaching adjunct and a lack of contextual framework influenced responses to these questions. For example, some educators had not been taught head and neck anatomy using ultrasound, so were unsure if it was practical or even useful to do so with their own students. This also may explain why the only procedure that educators were largely in favour of including was ultrasound-guided central venous access. There is a universal familiarity with this application among our clinical educators as our university has been teaching this as a mandatory residency course for several years.

A large number of neutral responses were also received for other clinical applications, despite 82% of educators favouring clinical ultrasound teaching to medical students. These included, but were not limited to, ultrasound guided nerve blocks, ruling out abdominal aortic aneurysms, focused abdominal sonography in trauma, and detecting pneumothoraces and thyroid masses. The focus group reported that ultrasound-guided procedures were higher-order skills not appropriate for undergraduate medical students early in their training. They also expressed concern with medical students independently ruling out potentially life-threatening conditions. Instead, they saw benefit in using ultrasound to augment their learning and reinforce their physical exam skills, such as correlating traditional palpation of the thyroid to its anatomical location with ultrasound. Educators also expressed a desire for standardized assessments to ensure competency and safe application of ultrasound. Such a tool has been developed and is undergoing external validation [22].

Although educators mostly agreed with the instruction of clinical applications, some educators disagreed with such an approach. There is a perceived safety risk in arming medical students with the ability to make diagnoses or perform procedures, especially when educators have no experience in these areas or perform traditional “blind” approaches to invasive procedures. Although the safety of completing such procedures with ultrasound guidance is well documented, it is understandable that educators would be nervous with it in the hands of a junior learner [23-25]. As the survey did not differentiate between clinical and preclinical medical students, the focus group also speculated that educators may have answered the survey with preclinical medical students in mind, and hence responded that some of these applications were not appropriate for more junior learners.

These findings suggest that clinical applications of ultrasound and ultrasound-guided procedures should be reserved for the more experienced clinical learners. Our data also supports that the non-clinical ultrasound applications are best suited to the junior medical learners. This has great implications for medical school curriculum development in North America and beyond, as the traditional approach has been to teach courses or select modules of courses originally designed for practicing clinicians. Teaching skills appropriate for the level of training is paramount. The traditional approach is not supported by our data and misses the opportunity to engage students in the basic medical sciences in a manner that is visual, informative, and enjoyable. Our data supports an approach more consistent with the extensive and well-established undergraduate curriculum at the University of South Carolina [1].

Our study is unique as it is the only comprehensive undergraduate ultrasound curriculum needs assessment that includes medical learners and educators. Unfortunately, our results are limited by our low response rate of 17.8%. We believe that this is partially due to the inclusive population we chose for our survey. We sought responses from all full-time faculty, many of whom are non-clinical faculty lacking experience with ultrasound. Our university was also undergoing a major curriculum change at the time of our survey, and requesting frequent input from students and faculty can introduce respondent fatigue and sampling bias.

Introducing an undergraduate ultrasound curriculum is a time and resource-intense undertaking, especially when it is longitudinally integrated. Until a standardized national curriculum is accepted, a needs assessment, such as the one described here, should be considered prior to implementation of such a complex intervention.

## Conclusions

An integrated undergraduate ultrasound curriculum composed of specific preclinical and clinical applications was deemed appropriate for our university following a comprehensive needs assessment. Other universities planning such curriculum should consider employing a needs assessment to provide direction for curriculum need and content.
